# Dengue Seroprevalence in the French West Indies: A Prospective Study in Adult Blood Donors

**DOI:** 10.4269/ajtmh.14-0211

**Published:** 2015-06-03

**Authors:** Maïna L'Azou, Janick Jean-Marie, Maël Bessaud, André Cabié, Raymond Césaire, Xavier de Lamballerie, Rémi Courbil, Pascale Richard

**Affiliations:** Global Epidemiology Department, Sanofi Pasteur, Lyon, France; EA 4537 Université des Antilles et de la Guyane; Centre d'Investigation clinique des Antilles et de la Guyane, CIC1424, Cayenne, Guyane; UMR_D 190 “Emergence des Pathologies Virales”, Marseille, France; Aix Marseille University, IRD French Institute of Research for Development, EHESP French School of Public Health; Service des Maladies Infectieuses et Tropicales, CHU de Martinique, Fort-de-France, Martinique; Laboratoire de Virologie, CHU de Martinique, Fort-de-France, Martinique; Etablissement Français du Sang, Pointe à Pitre, Guadeloupe; Etablissement Français du Sang, Hôpital Pierre Zobda Quitman, Fort-de-France, Martinique

## Abstract

Using an anti-dengue immunoglobulin G (IgG) indirect enzyme-linked immunosorbent assay, seroprevalence was determined among 783 adult blood donors in the French Caribbean islands of Guadeloupe and Martinique in 2011. Overall, 93.5% [91.5; 95.1] samples were positive for dengue antibodies, 90.7% (350 of 386) in Martinique and 96.2% (382 of 397) in Guadeloupe. Only 30% of these adults recalled having had dengue disease before. Serotype-specific neutralization assays applied to a subset of IgG-positive samples indicated that a majority (77 of 96; 80%) reacted to the four serotypes. These seroprevalence findings are the first reported for Guadeloupe and Martinique and are consistent with the dengue epidemiology in these territories.

The resurgence of dengue disease in the Americas in recent decades is well documented. In the French Caribbean islands of Guadeloupe and Martinique dengue is endemic with the co-circulation of more than one serotype. Peaks in disease incidence occur in the wet season (June to November) and affect thousands of people. In Martinique (population: 396,000) the number of suspected cases of dengue was 24,000–26,500 during an epidemic in 2001–2002, 14,500 during an epidemic in 2005–2006, 18,000 in 2007–2008, and 40,000 in 2010.^1^–^3^ Similarly, in Guadeloupe (population: 402,000), 11,500 suspected cases were reported during the 2005–2006 epidemic, 19,000 during the 2007 epidemic, and more than 43,000 suspected cases in 2010.^1^,^2^,^4^,^5^

The burden of dengue virus infections is not known in these islands because the majority of dengue virus infections are thought to be asymptomatic. Evidence of dengue virus infection is provided by studies of the prevalence of anti-dengue immunoglobulin G (IgG) antibodies in endemic areas. Published reports show seroprevalence rates of 98% among adults and 56% among children < 10 years of age in the Dominican Republic in 2002, 93% among a broad age range of residents in southern Grenada in 1996, 94% among pregnant women in Trinidad, and 92% of adults in Puerto Rico in 2006.^6^–^9^ Another study conducted between 2003 and 2006 among suspected dengue cases of all ages in Jamaica found a seroprevalence rate of 51%.^10^ A recent study among pregnant women in 10 Caribbean islands (not including Guadeloupe or Martinique) found dengue seroprevalence rates of 80–100% in all but one of the islands.^11^ No such data exist for Guadeloupe or Martinique.

We estimated the seroprevalence of antibodies against dengue in samples collected between May 2011 and June 2011 from adults at blood donor centers. On each Island blood donations are collected either at the central French Blood Establishment center, or in mobile donor units. Participants were therefore recruited from throughout the islands. Each volunteer orally answered a short series of demographic questions to document age, gender, country of birth, and place of residence, and their dengue history: recollection of having had dengue, number of infections, and year of last infection.

We screened 831 volunteers. Samples from 14 participants were excluded from the study as a result of missing documentation, and samples were not, or could not be, collected from a further 34 individuals for practical reasons. Samples analyzed were therefore collected from 783 individuals: 386 in Martinique and 397 in Guadeloupe. Participants were 18–70 years of age (mean age of 38.3 years and median age of 38.0 years), and 47% were males. Inclusion criteria were eligibility to give blood according to French regulations and residence on the island for at least 1 year. The study was approved by the French South West and Overseas ethics committee (Comité de Protection des Personnes Sud Ouest et Outre-Mer III) and was conducted in accordance with good clinical practices. Participants gave written informed consent. Data were analyzed using Stata v10.0 software (Stata, College Station, TX).

Sera were tested using a dengue IgG indirect enzyme-linked immunosorbent assay (ELISA) (Panbio, Queensland, Australia) at the Etablissement Français du Sang, Martinique. Overall, 732 of 783 (93.5%) samples (95% confidence interval [CI]: 91.5; 95.1) (Table 1) were positive for dengue antibodies, 350 of 386 (90.7%) in Martinique, 382 of 397 (96.2%) in Guadeloupe. To our knowledge, there is no evidence to suggest the current or past circulation of other flaviviruses among humans on these two islands, where there is no yellow fever vaccination program. Our results are therefore likely to closely reflect the prevalence of dengue. In comparison, only 30% of the enrolled participants recalled having had dengue disease. This discrepancy highlights the high proportion of dengue virus infections that are asymptomatic or misattributed to other etiologies caused by the mildness or non-specificity of symptoms. Seroprevalence seemed greater in those > 40 years of age than in younger age groups, but did not increase beyond that age (Table 1; post-hoc χ^2^ test, *P* < 0.001.). However, this study was not designed to detect differences in seroprevalence by age. Seroprevalence was also significantly different between those born on the islands, compared with those who moved to the islands (Table 1; χ^2^ test, *P* < 0.001). Further interpretation of these results is limited because the duration of residence in the Caribbean, and where childhood was spent was not documented.

A random subset of 96 (13%) of the IgG-positive samples were selected for serotyping at the Center Hospitalier Universitaire, Marseille, France, using an ELISA-format microneutralization test described elsewhere, which has been shown to be similar monotypic responses to the standard serotype-specific plaque reduction neutralization test (PRNT) assay in serum post primary infection.^12^ Briefly, viruses were grown in 96-well plates in Vero E6 monolayers in the presence of 2-fold serial dilutions of serum ranging from 1/40 to 1/2,560. Seven days post-infection, supernatants were removed; cells were fixed with 4% paraformaldehyde and permeabilized with 0.5% Triton X-100. Virus proteins were detected spectrophotometrically using pan-flavivirus anti-NS5 antibodies from hybridoma H86.13 B4A supernatant, commercially available horseradish peroxidase (HRP)-conjugated anti-mouse secondary antibodies and 3,3′,5,5′-Tetramethylbenzidine (TMB) peroxidase substrate. For each serum and virus, the neutralization titer was the reciprocal of the highest serum dilution that inhibits the virus growing. Negative controls were performed using samples from our collection, sampled from patients who had never traveled to dengue-endemic countries. The dengue 1–4 viruses used in the assay were respectively H/IMTSSA/98/060, H/IMTSSA-MART/98-703, H87, and Dak HD 34 460.^13^–^16^ The randomly selected sera were from patients with a mean age of 37.6 years and a median age of 37.0 years. Of these samples 77 of 96 (80%) were seropositive to all four serotypes (titer ≥ 40), 11 of 96 (11%) were seropositive to three serotypes, 3 of 96 (3%) were seropositive to one serotype, and 5 of 96 (5%) were seronegative, with a titer below the detection limit of 40. Neutralizing antibodies against serotypes 1, 2, 3, and 4 were detected in 85%, 89%, 93%, and 82% of samples, respectively. The geometric mean neutralizing antibody titers were higher in Guadeloupe than in Martinique (Figure 1), for DENV-1, -2, and -3 (Wilcoxon test, *P* < 0.05). For all serotypes geometric mean titers appeared higher among older donors (≥ 37 years of age): 602, 2,269, 1,704, 1,339, against serotypes 1–4, respectively, compared with 240, ,068, 733, and 991 among those ≤ 36 years of age (Figure 1). Furthermore, 93% (42 of 45) of donors aged 37 or more were seropositive for all four serotypes, compared with 66% (31 of 47) among younger donors (age was missing in four cases). Although the neutralization assay does not distinguish between homotypic and heterotypic dengue neutralization responses in case of sequential infections by different dengue virus serotypes, these results suggest that most of our study population had been exposed to more than one dengue virus serotype.^17^ In a 2006 study in Puerto Rico, 96% of anti-dengue IgG positive samples were reported to have shown evidence of secondary infection, including 10% that were considered to have probably been recent infections.^9^

**Figure 1.**
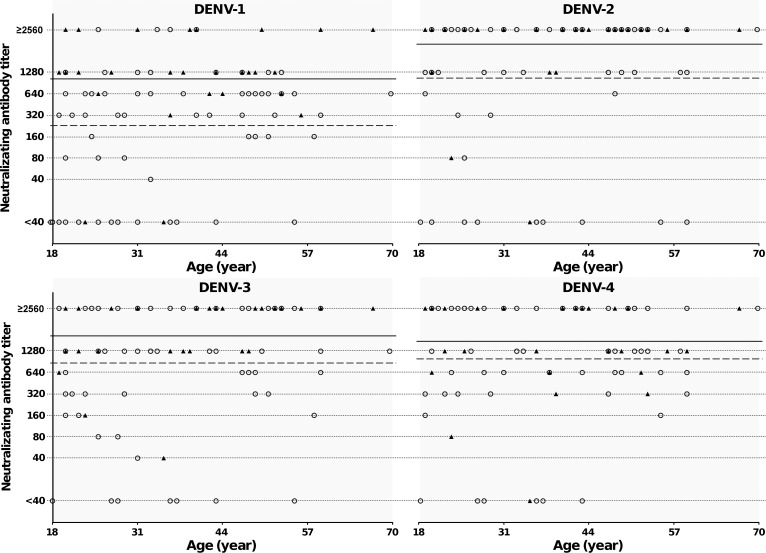
Scatter plots of end-point antibody titers versus age of the blood donors from Martinique (○) and Guadeloupe (Δ) in 2011 (*N* = 89). Antibody titer is defined as the reciprocal of the highest serum dilution that inhibits virus growing.^10^ For each dengue virus (DENV) serotype, the dashed and solid lines indicate the geometric mean of antibody titers of sera from Martinique and Guadeloupe, respectively; geometric means were calculated by assigning a value of 10 to titers < 40 and a value of 5,120 to titers ≥ 2,560.

Our seroprevalence findings, in samples collected mid 2011, are consistent with the epidemiology as all dengue virus serotypes have been identified in Martinique and Guadeloupe during recent epidemics. In Martinique, DENV-3 and DENV-2 both circulated in 2001, DENV-4 and DENV- 2 circulated in 2005, and DENV-1 and DENV-4 circulated in 2010.^2^–^4^ In each case previously mentioned, the first serotype listed was the predominant epidemic strain. In Guadeloupe an epidemic of DENV-4 occurred in 2005, with DENV-2 co-circulating. DENV-2 then predominated from 2006–2007, before being replaced in 2008 by DENV-1 as the predominant strain with DENV-2 co-circulation and sporadic cases of DENV-3.^4^,^5^ The DENV-1 was also predominant in 2010, with DENV-4 co-circulation.^4^,^5^ On both islands, the 2010 epidemic was particularly intense, with 10% of the population having suspected dengue. The epidemic in Martinique spanned 36 weeks from February to October 2010 and counted 40,000 suspected cases and in Guadeloupe it spanned 47 weeks, from November 2009 to October 2010 and counted more than 40,000 suspected cases.^3^–^5^

The age-distribution of dengue disease on Martinique and Guadeloupe has not been reported in detail. Although our study only included adults, our finding that 87% of 18- to 19-year-olds were anti-dengue IgG seropositive suggest that dengue is particularly prevalent during childhood or adolescence. Understanding the seroprevalence and disease burden in children would help to further our understanding of the epidemiology of dengue disease in these islands overall. In summary, our study confirmed that dengue viruses circulate in Martinique and Guadeloupe at levels comparable to those seen on neighboring islands. In each age cohort in our study population, 87–97% were dengue seropositive, with evidence of more than one dengue virus infection, yet only 30% remembered having had dengue.

## Figures and Tables

**Table 1:** Seroprevalence of anti-dengue IgG in adult blood donors in Martinique and Guadeloupe according to age and birthplace in 2011 (*N* = 783)

	Seroprevalence % (n/*N*)	Confidence intervals (95%) (exact method)
18–70 years	93.5% (732/783)	[91.5; 95.1]
18–19 years	86.7% (26/30)	[69.3; 96.2]
20–29 years	91.7% (176/192)	[86.8; 95.2]
30–39 years	90.0% (171/190)	[84.8; 93.9]
40–49 years	96.9% (188/194)	[93.4; 98.9]
50–59 years	96.6% (142/147)	[92.2; 98.9]
60–70 years	96.7% (29/30)	[82.8; 99.9]
Born in the French West Indies	97.4% (551/566)	[95.7; 98.5]
Born in Continental France	82.2% (152/185)	[75.9; 87.4]

IgG = immunoglobulin G.
